# Supplementary material for: Matrix WaveTM System for Mandibulo-Maxillary Fixation—Just Another Variation on the MMF Theme? Part I: A Review on the Provenance, Evolution and Properties of the System
Source: Craniomaxillofac Trauma Reconstr. 2025 Jul 12;18(3):32. doi: 10.3390/cmtr18030032 (PMC12286117; doi:10.3390/cmtr18030032)
Supplement: Supplementary file 1 [file cmtr-18-00032-s001.zip › cmtr-3499161-Supplementary.pdf]

Supplemental Electronic Content

for

Cornelius CP, Liokatis PG, Doerr T, Damir M, Fusetti S, Rasse M, Gellrich NC, Heiland M, Schubert W, Buchbinder D. Matrix Wave™ System for mandibulo-maxillary fixation - just another variation on the MMF theme ? – **Part I**: A Review on the Provenance, Evolution and Properties of the System. *Craniomaxillofac Trauma Reconstr*. 2025, *18*, 32

• *Text S1*

• *Cautionary Note to Figure 17*

Cautionary Note: The sole purpose of these figures is to illustrate the in-situ-bending process of the Matrix Wave™ plate for fragment reapproximation/reduction. It is not intended to address any bio-mechanical implications and problems of (para-) median / (para-) symphyseal mandibular fractures. These fractures are prone with widening of the posterior mandible and the face, because of lateral displacement of the rami. The risk of widening increases with combined (uni-/bilateral) fractures of the angle region and/or condylar process. The expansion of the transverse distance between the gonions is directly correlated with the gap of the lingual cortex at the symphyseal fracture site. The anatomic reduction of symphyseal fractures requires control of accurate interfragmentary closure and adequate stabilization of the preinjury anatomy (Ellis and Tharanon 1992 [43], Chen et al 2016 [44], Gerbino et al 2009[45]).

References:

43. Ellis E, 3rd, Tharanon W. Facial width problems associated with rigid fixation of mandibular fractures: case reports. *J Oral Maxillofac Surg*. Jan 1992;50(1):87-94. doi:10.1016/0278-2391(92)90206-f

44. Chen S, Zhang Y, An JG, He Y. Width-Controlling Fixation of Symphyseal/Parasymphyseal Fractures Associated With Bilateral Condylar Fractures With 2 2.0-mm Miniplates: A Retrospective Investigation of 45 Cases. *J Oral Maxillofac Surg*. Feb 2016;74(2):315-27. doi:10.1016/j.joms.2015.09.030

45. Gerbino G, Boffano P, Bosco GF. Symphyseal mandibular fractures associated with bicondylar fractures: a retrospective analysis. *J Oral Maxillofac Surg*. Aug 2009;67(8):1656-60. doi:10.1016/j.joms.2009.03.069

• *Text S 2*

• Biomechanical effects – Vertical level of intermaxillary connection

In an overly simplistic way tooth- or bone-supported MMF application can be understood as a system of levers (Fig. S2). Within such a system positioning fasteners at the vertical height of the tooth necks or gingival margin, is counterproductive to maintaining balanced occlusal contact.

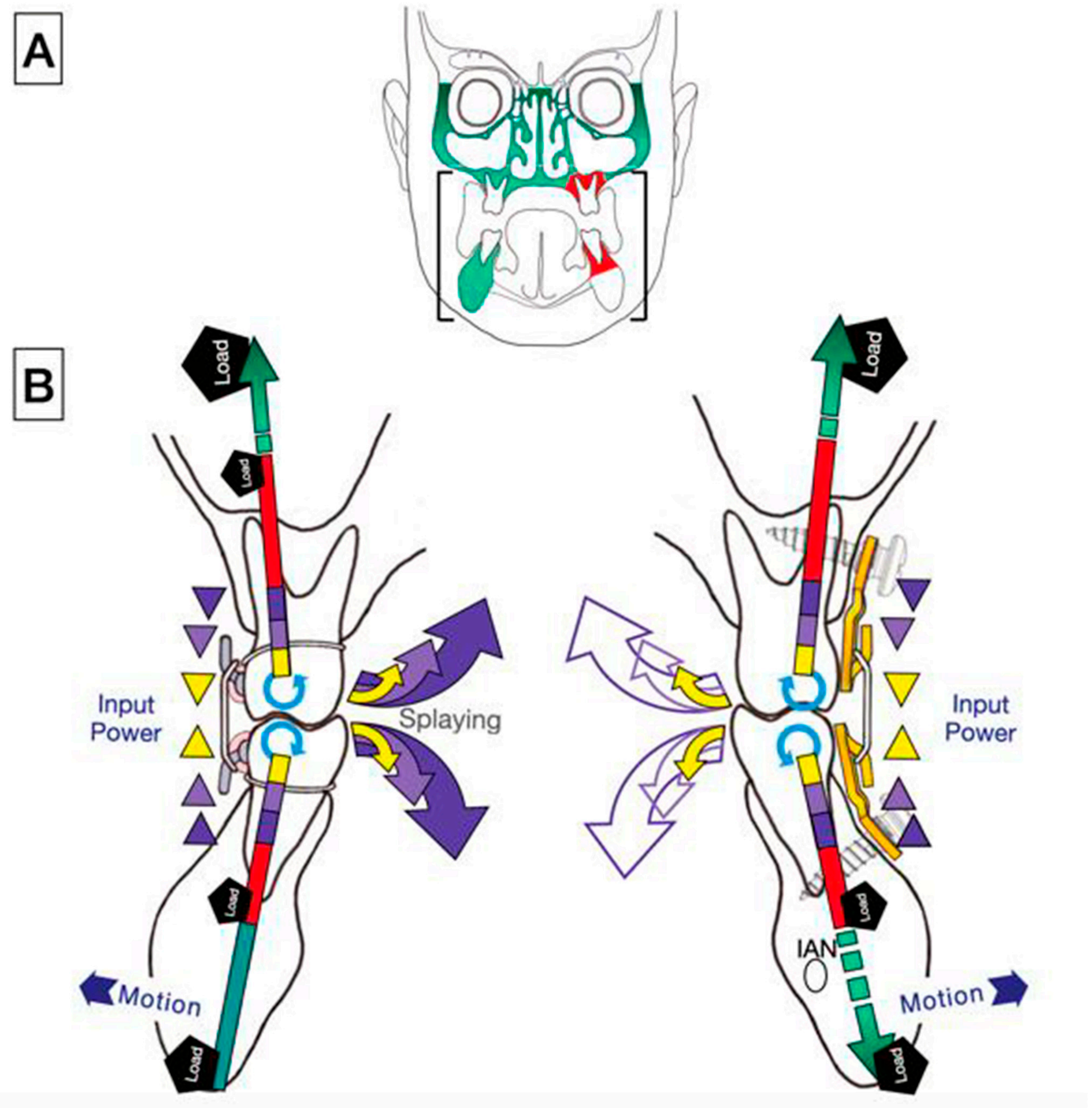

• **Figure S2.** Biomechanical implications of fasteners for intermaxillary connection.
Fig. S2 A (top): Coronal cross section of an adult head in the 1st molar region. Alveolar process fracture in left maxilla and mandible (red) Le Fort I, II, III fracture planes and mandibular body with fully intact cross section (serially darkening green).
Fig. S2 B (bottom left): Detail – Intermaxillary fixation (MMF) via arch bar hooks at the left buccal side can be modeled as two vertically opposed class 3 levers. Both levers are pivoted at an identical fulcrum that corresponds to the occlusal contacts between the cusps and fossae of the mandibular and maxillary teeth. The length of the lever arms is graded into three additive subdivisions by color-coded stacked rectangles in unison with equilateral triangles alongside having the following connotations – yellow represents a short arm according to fasteners located in juxtaposition of the dental crown equator; purple means an elongated arm due to transposition of the fastener towards the tooth neck; violet blue equates an alignment of the fasteners next to the attached gingiva or seated further inside the vestibulum. The load arms consist of two consecutive components - the red bar refers to the section of the load arm that meets with the height of the alveolar process fractures as depicted in the top diagram **(A)**; the green part bears upon expanded large fragments in the maxillae or mandible. The input power (ef- fort) is generated by the magnitude of the tensile forces of the intermaxillary linkage (wire ligatures, rubber loops).The resistance to load or output power (i.e. lateral motion) coincides with the hori- zontal course of the fracture lines and the degree of fragmentation. Eventually several mutually dependent factors are at play for the tipping of the fragments all together with the degree of angu- lation of the lingual occlusal splaying.
Fig. S2 B (bottom right): The effects of intermaxillary fixation (MMF) via MWP tie-up-cleats are subject to the same parameters as outlined by way of hooks. The vertical level of the MWP and its hooks can be easily and firmly adjusted over a wide vertical range (indicated by the equilateral triangles). To respect the inferior alveolar canal/nerve is absolutely mandatory when choosing a low mandibular screw insertion site.

Source/origin:
Draft – C.P. Cornelius. Idea based on Fig. 176 from Spiessl B, Internal Fixation of the Mandible – A Manual of AO/ASIF Principles, Part II, Internal Fixation of Fresh Fractures, 4. Surgical Approaches, 4.2. Principle of Combined Fracture Treatment. p. 169, Berlin Heidelberg New York – Springer-Verlag 1989; and: Manson PN, Facial fractures, In: Grabb and Smith's Plastic Surgery 5th Edition, Aston SJ, Beasley RW, Thorne CHM (Eds.). Chapter 34, p.402, Fig.32 Philadelphia – Lippincott Raven Publishers 1997 or Manson PN, Shack RB, Leonard LG, Su CT, Hoopes JE. Sagittal fractures of the maxilla and palate. Plast. Reconstr Surg. 1983;72(4):484-9. doiI: 10.1097/00006534-198310000-0001

As the vertical location moves upwards (maxilla) or downwards (mandible), respectively the effectivelength of the lever arms increases. Reciprocally the length of the load arms which correspond to the height of the bone fragments is shortened.

In the worst case scenario with multifragmentation of the alveolar process and in due course downsized load arms amplify the input forces and can generate a tilting moment strong enough to splay the lingual occlusion. Clinically this minimal and even moderate occlusal opening movements may go unnoticed because closely maintained occlusal contacts at the buccal side may obscure the assessment of the lingual/palatal aspect. Pedemonte et al. (2019) [51] examined the gapping of a midline palatal fracture after reduction by means of MMF in a laboratory study on acrylic dental upper jaw models. In keeping with the effects caused by long lever arms or "vector forces far away

from the center of resistance at the level of the occlusal contacts" a four point MMF using pairs of specialized bone screws and wire cerclages resulted in major splaying and tipping of the palatal halves. Direct interdental wiring (by two pairs of embrasure wires) or intermaxillary wired Erich Arch bars in closer proximity to the occlusal pivot points eliminated or reduced the splay and tilt between the relatively small fragments.

In contrast large fragments after proper reduction – for instance le Fort I, II, III or extended non-condylar mandibular fragments – yield more stability and enough resistance (load) to withstand heavy output forces and subsequent infero- and supero-lateral displacement of the mandible and/or maxillae. With MMF installation there is ample support for applying elastic couplings to achieve an even distribution of forces and a balanced occlusion instead of wire ligatures. In particular the strong pull and excessive twisting of wire loops could be disruptive since this builds up a disproportionate input power.

Reference:

51. Pedemonte C, Valenzuela K, González LE, Vargas I, Noguera A. Types of intermaxillary fixation and their interaction with palatine fracture reduction. *J Oral Maxillofac Surg*. Oct 2019;77(10):2083.e1-2083.e8. doi:10.1016/j.joms.2019.06.006
